# Optical Measurements and Theoretical Modelling of Excitons in Double ZnO/ZnMgO Quantum Wells in an Internal Electric Field

**DOI:** 10.3390/ma14237222

**Published:** 2021-11-26

**Authors:** Janusz Andrzejewski, Mieczyslaw Antoni Pietrzyk, Dawid Jarosz, Adrian Kozanecki

**Affiliations:** 1 Department of Experimental Physics, Faculty of Fundamental Problems of Technology, Wrocław University of Science and Technology, ul. Wybrzeże Wyspiańskiego 27, 50-370 Wrocław, Poland; 2 Institute of Physics, Polish Academy of Sciences, Aleja Lotnikow 32/46, 02-668 Warsaw, Poland; pietrzyk@ifpan.edu.pl (M.A.P.); djarosz@MagTop.ifpan.edu.pl (D.J.); kozana@ifpan.edu.pl (A.K.); 3 Institute of Physics, College of Natural Sciences, ul. Pigonia 1, 35-959 Rzeszow, Poland

**Keywords:** semiconductors, ZnO, photoluminescence, excitons, effective-mass theory

## Abstract

In this paper, the photoluminescence spectra of excitons in ZnO/ZnMgO/ZnO double asymmetric quantum wells grown on a–plane ${\mathrm{Al}}_{2}O_{3}$ substrates with internal electric-field bands structures were studied. In these structures, the electron and the hole in the exciton are spatially separated between neighbouring quantum wells, by a ZnMgO barrier with different thickness. The existence of an internal electric field generates a linear potential and, as a result, lowers the energy of quantum states in the well. For the wide wells, the electrons are spatially separated from the holes and can create indirect exciton. To help the understanding of the photoluminescence spectra, for single particle states the 8 k·p for wurtzite structure is employed. Using these states, the exciton in the self-consistent model with 2D hydrogenic 1s–like wave function is calculated.

## 1. Introduction

In the last decade, there has been a rapid progress in studies of wide bandgap semiconductors, with zinc oxide (ZnO) drawing special attention for its unique and very promising physical properties. High electron mobility, high thermal conductivity and high exciton binding energy (60 meV at room temperature) [1] are some of the most important properties that can be utilized in optoelectronics, piezoelectric devices, transparent and spin electronics and in chemical sensor applications. Apart from its wide bandgap (∼3.3 eV), the ZnO layers are characterized by their high optical transmission in the visible spectrum [2], as well as by easily controllable electrical parameters depending on the deposition conditions. Zinc oxide has been an intensively researched material for many years, mainly due to the fact that it is considered as an alternative, and hence competitive, solution to gallium nitride. Currently, blue LED diodes are produced on the basis of GaN and other nitrides (InN, AlN). The use of oxides instead, including ZnO, would lead to a significant reduction in the device manufacturing cost. Transition metal dichalcogenides, especially those with metals from group 4 of the periodic table because of energy bandgaps in the range 0.2–2 eV, could not be a direct competitors to the blue-emitting devices.

Other ZnO superior properties over other compound semiconductors, including high melting temperature (2248 K), biocompatibility, low toxicity and low cost, open new perspectives for application in biological sensors or high power laser structures or field effect transistors. Furthermore, the possibilities of fabricating nanostructures from ZnO or ZnMgO, such as e.g., nanocolumns, make it an interesting object for basic research.

The most common orientation of crystal growth for oxide is in a polar direction c–plane (0001). This is the case of growth on silicon [3] and sapphire substrates with a–plane ($11\bar{2}0$) [4] or c–plane (0001) [5] crystallographic direction. In this direction, a strong built-in electric field is present due to the spontaneous and piezoelectric polarization. Quantum states in bilayer systems are sensitive to the electric field applied along the growth direction of the structure. The spontaneous electric field strength across the ZnO and ZnMgO unit cells is proportional to the Mg concentration. The difference in polarity is opposite on either side of the QW, resulting in an electric field across the QW. One of the consequences of the presence of electric fields is a quantum confined Stark effect, resulting in spatial separation of electron and hole wave functions (WF) in quantum wells, and lowering the probability of radiative transitions—a very inconvenient effect for optoelectronic applications. The electric field modifies the WF confinement in each well. Instead of spreading in each of the wells, the carriers become essentially localized by triangular wells. As a result, a red-shifting of the exciton transition energy, as well as decreasing of the exciton oscillator strength and binding energy are observed. In quasi two-dimensional structures, two kinds of the Stark effect are possible. The electric field can be applied either parallel to the growth axis Z, or perpendicular to it. If the electric field is applied along the z–direction, the electrons and holes will be pulled into different quantum wells due to their opposite charges, and form spatially indirect exciton (IX). An IX is a bound pair of an electron and a hole in separated by a thin tunnel barrier [6]. Electron and hole will relax into their respective wells by tunnelling through the narrow barrier layer. IX in coupled quantum wells form a unique system for development of novel optoelectronic devices.

Their close proximity allows them to interact and form a bound state. The energy levels, binding energy and Bohr radius of IX can be calculated by different methods [7,8]. The spatial separation between the electron and hole layers allows one to control the overlap of electron and hole WF and engineer structures with long IX lifetimes [9].

In this paper, we probe the evolution of IXs peaks in ZnO/ZnMgO DQWs structures. The binding energy of IXs is smaller than that of excitons in bulk ZnO, however it is large enough to make the IXs stable at room temperature [10]. The most common type of substrate used for the growth of all oxide structures is a–plane sapphire. Due to the minimal lattice mismatch (∼1.5%), this substrate allows for growing planar structures with excellent crystal quality. The presence of an electric field of magnitude 0.9 MV/cm was reported [11], indicating that the fields generated this way may be particularly strong.

Asymmetric double QWs separated by a narrow potential barrier provide an ideal platform for studying coupling between spatially separated excitons. The barrier must be thin enough to allow tunnelling of carriers between the two QWs. The WF originating from one QW extends into the other QW, which means that the electron or hole lies in both QWs simultaneously, but with different quantum probabilities. The coupled double QWs in electric fields are especially important due to possible formation of indirect excitons in such a system. For the structure with thick barrier due to the spatial separation of the particles, (small overlap of the single-particle wave functions), different physical properties are expected for the excitons in comparison to the structure with very narrow barrier. The barrier width decides on the importance of the excitonic effects. Therefore, we choose for our study three structures of ZnO/ZnMgO double quantum wells: two with a very narrow ZnMgO barrier and different widths of QWs in order to observe IXs, and the third one with a wide barrier as the reference sample with weak tunnel coupling between the two wells.

The theory used in this article is the 8 k·p wurtzite model for single particle states, and based on these states the exciton is calculated. By partial integration of the excitonic wave function, some additional potentials appear and, within the so-called self-consistent field theory, this potential is calculated.

## 2. Experiment and Theory

### 2.1. Growth Method

The samples that we investigated are structures grown by molecular beam epitaxy (MBE) on a–plane ${\mathrm{Al}}_{2}O_{3}$ substrate. Conventional Knudsen-cells were used for the evaporation of Zn (6N) and Mg (5N) pure elements. Before the growth process, the ${\mathrm{Al}}_{2}O_{3}$ substrates were chemically and thermally cleaned. The substrates were oxidized in the growth chamber at a temperature of 700 °C for 30 min. During the growth process, the oxygen flow was kept at 2.6 sccm and the rf power of oxygen plasma was fixed at 400 W. The structures were grown at a temperature of 500 °C. The growth temperature was controlled by a thermocouple placed behind the substrate. The Zn flux was adjusted to about $8\times 10^{-7}$ Torr and Mg to about $2\times 10^{-8}$ Torr.

First, a ZnMgO buffer layer was grown on the substrate up to the thickness of about 300 nm. On top of the ZnMgO barrier material, a structure with an asymmetric double quantum well with different barrier width (these structures are marked as #1, #2 and #3) was deposited. Finally, the whole structure was covered with a 20 nm ZnMgO cap layer. The thicknesses of the nanostructures were estimated based on growth conditions. The growth rate was controlled in situ with laser reflectometry technique. The growth of the structures was monitored in situ by RHEED.

### 2.2. Optical Measurement

The optical properties of the QWs structure were investigated by photoluminescence (PL) using a 302.4 nm line (20 mW) of an ${\mathrm{Ar}}^{+}$ ion laser as the excitation source. The PL signal was dispersed with 0.75 m focal length Horiba monochromator and detected with Hamamatsu R375 photomultiplier and amplified by EG&G D7265 Lock-in.

### 2.3. The Theoretical Model

The energy levels of an exciton in a QWs can be described in terms of the effective mass approximation [12,13]. The total appropriate Hamiltonian $H_{eff}$, representing the exciton, consists of the three terms. The first two are single particle (SP) one dimensional Hamiltonians $H_{e}\left(z_{e}\right)$ and $H_{h}\left(z_{h}\right)$ describing the separate motions along the growth direction of the electron and hole, respectively. The last term is the heart of the exciton—it is described by the interaction term $H_{rel}$, representing their relative motion and arising from the Coulombic interaction term. Thus

(1) $$H_{eff}=H_{e}\left(z_{e}\right)+H_{h}\left(z_{h}\right)+H_{rel}.$$

This equation could only be written down when Hamiltonians $H_{e}\left(z_{e}\right)$ and $H_{h}\left(z_{h}\right)$ are not degenerating [14,15,16,17]—we ignore the center of mass motion because it is related e.g., to inhomogeneous broadening and Stokes shift between photoluminescence and absorption.

The solution to the exciton Hamiltonian $H_{eff}$ is obtained via a variational approach employing a trial exciton WF in the following form [12,13,18,19]—separable in growth direction and X–Y directions (QW plane):

(2) $$\Psi \left(r_{e},r_{h}\right)=\psi_{e}\left(z_{e}\right)\psi_{h}\left(z_{h}\right)\varphi_{e-h}\left(\rho_{e},\rho_{h},z_{e},z_{h}\right),$$

where $r_{e}=\left(x_{e},y_{e},z_{e}\right)=\left(\rho_{e},z_{e}\right)$ and $r_{h}=\left(x_{h},y_{h},z_{h}\right)=\left(\rho_{h},z_{h}\right)$ are the electron and hole coordinates, respectively.

The multipliers $\psi_{e}\left(z_{e}\right)$ and $\psi_{h}\left(z_{h}\right)$ are the eigen functions of the effective SP Schrödinger-like equation for the electron and the hole, respectively. The last multiplier in Equation (2)—$\varphi_{e-h}\left(\rho_{e},\rho_{h},z_{e},z_{h}\right)$—is the trial WF describing the relative motion of the electron and hole and we choose normalized 2D hydrogenic 1s–like wave-function, containing only one variational extension parameter λ:

(3) $$\varphi_{e-h}\left(\rho_{e},\rho_{h},z_{e},z_{h}\right)=\varphi \left(\rho \right)=\frac{2}{\lambda }\exp\left(-\rho /\lambda \right),$$

where $\rho =\sqrt{{\left(x_{e}-x_{h}\right)}^{2}+{\left(y_{e}-y_{h}\right)}^{2}}$ measures the relative electron-hole distance in the transverse direction.

Even if we limit ourselves to the exciton WF according to Equations (2) and (3), the additional potential appears [20,21,22], which comes from perpendicular motion and Coulomb interaction of the electron and hole:

(4) $$W\left(z\right)=\int_{0}^{\infty }\rho d\rho \left(\frac{\hbar^{2}}{2\mu }{\left(\frac{d\varphi_{e-h}\left(\rho \right)}{d\rho }\right)}^{2}-\frac{e^{2}}{4\pi \varepsilon \sqrt{\rho^{2}+z^{2}}}\right).$$

This W(z) potential has to be added to the band edge potentials for electron and hole. The resulting one–dimensional effective Schrödinger-like equations for electron and hole WF, respectively:

(5) $$\left(H_{e}\left(z\right)+W_{h}\left(z\right)\right)\psi_{e}\left(z\right)=E_{e}\psi_{e}\left(z\right),$$

(6) $$\left(H_{h}\left(z\right)+W_{e}\left(z\right)\right)\psi_{h}\left(z\right)=E_{h}\psi_{h}\left(z\right)$$

must be solved together since the Coulomb term

(7) $$W_{\nu }\left(z\right)=\int dz^{\prime }{\left|\psi_{\nu }\left(z^{\prime }\right)\right|}^{2}W\left(z-z^{\prime }\right)\;\mathrm{for}\;\nu =e,h$$

couples the electron $\psi_{e}\left(z\right)$ and hole $\psi_{h}\left(z\right)$ WF.

The Schrödinger-like equation for the radial motion can be written as follows:

(8) $$\left(-\frac{\hbar^{2}}{2\mu }\left(\frac{1}{\rho }\frac{\partial }{\partial \rho }\left(\rho \frac{\partial }{\partial \rho }\right)\right)+V\left(\rho \right)\right)\varphi_{e-h}\left(\rho \right)=E_{B}\varphi_{e-h}\left(\rho \right),$$

where V(ρ) is the effective in-plane Coulomb potential, which is expressed as

(9) $$V\left(\rho \right)=-\frac{e^{2}}{4\pi \varepsilon }{\;\int \int \;}_{-\infty }^{\infty }\frac{|\psi_{e}\left(z_{e}\right){|}^{2}{\left|\psi_{h}\left(z_{h}\right)\right|}^{2}}{\sqrt{\rho^{2}+{\left(z_{e}-z_{h}\right)}^{2}}}dz_{e}dz_{h}.$$

Solving the set of Equations (5), (6) and (8) by the method of successive iterations [23,24], the total energy of the exciton could be calculated. This is a self-consistent treatment in which the single particle (Equations (5) and (6)) and “excitonic” radial multipliers (Equation (8)) of the excitonic WF (Equation (2)) are solved. The renormalization of the confining electron and hole potentials via the Equation (4) also results in self-consistent treatment of the Coulomb interaction and provides more accurate values for binding energies.

For SP Hamiltonian, the 8 k·p model for structure of wurtzite [25,26] is used with accounts for strain effects, strain-induced piezoelectric polarization and spontaneous polarization. All physical equations were numerically solved using the finite difference method [27]. The Luttinger-like parameters and deformation potentials are taken from [28], elastic, and polarization parameters, lattice constants from [29] and the energy band gap from [30], valence band splittings from [31] and band offset from [32].

### 2.4. Calculation Methodology

Using the geometry parameters of the sample like cap layer, QWs width, barrier and buffer widths and magnesium composition in each layer the electric field is calculated [26]. After that, using exactly the same geometry, the confinement potential is obtained by adding a valence band offset, the calculated electric field, and a strain related modification of the energy band. The SP energy and WF for electron and hole are calculated for that confinement potential. For all pairs of single particle energy levels of electron and hole, the exciton total energy and its binding energy is calculated as described above. Using the electron and hole WF multipliers of the exciton, the oscillator strength is calculated for comparison with the SP transitions. From all calculated excitons, only the ones with the strongest oscillator strength are considered.

## 3. Results

We studied the asymmetric system consisting of QWs of different widths (4 and 5 nm) separated by a 12 nm (structure #1), 2.5 nm barrier (structure #2) and sample with 4 and 2 nm QWs and 2.5 nm ZnMgO barrier (structure #3). The QWs are wide enough for the Stark effect to be observed. The internal electric field is directed in the growth direction c. As a result, the quantum well structures are tilted.

The theoretical model described above allows the calculation of not only SP energy transitions within 8 k·p wurtzite model, but also direct and indirect exciton in asymmetric double QWs. For both types of this exciton, the exciton binding and transition energy is calculated in a self-consistent manner.

### 3.1. Structure #1: 4 nm and 5 nm ZnO QWs with 12 nm ZnMgO Barriers

As the reference structure, the structure with 4 and 5 nm QWs and 12 nm wide ZnMgO barrier was first given. In this asymmetric system, the energies of the direct excitons of the narrow and wide wells are well separated, which considerably facilitates the interpretation of the optical spectra. The calculated electric fields have values 0.0068 MV/cm and 0.25 MV/cm in the barrier and in both QWs, respectively.

**Figure 1:**
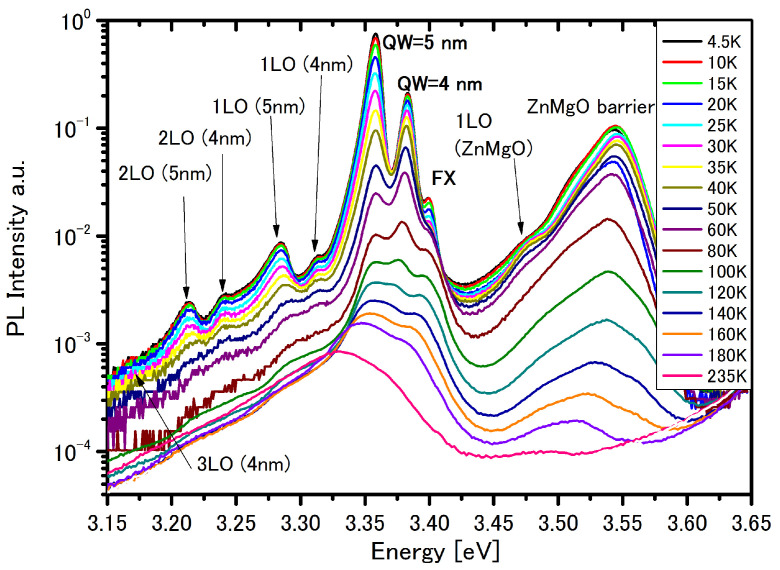
PL spectra at different temperature for structure #1.

Figure 1 shows the PL spectra of the structure #1 with the double quantum wells with 4 and 5 nm widths, with the barrier of about 12 nm thickness in different temperatures. The high energy peak at 3.544 eV corresponds to the PL emission of the ZnMgO cap/barrier layer with Mg content of about 10%. Luminescence of the ZnMgO barrier originates in recombination of localized excitons to neutral donors $D^{0}X$ [33]. The broadening of the ZnMgO emission is due to fluctuation effects, arising from variation in the Mg composition and lattice disorder in the ZnMgO alloy [34]. Emission from the ZnMgO barrier layer is not observed at temperatures above 235 K. We observed phonon replicas from the ZnMgO barrier at 3.472 eV.

The transitions denote that 1 LO, 2 LO and 3 LO are longitudinal phonon (LO) replicas, since their position and energy distances match the 72 meV intervals counting from the position of the 4 nm and 5 nm QWs.

The emission peak observed at 3.358 eV and 3.383 eV contributes to SP transitions in 5 nm and 4 nm QWs, respectively—Table 1.

In Table 2 some properties for various type of excitons calculated within 8 k·p self-consistent model described above which may be observed in the considered structure are presented.

**Figure 2:**
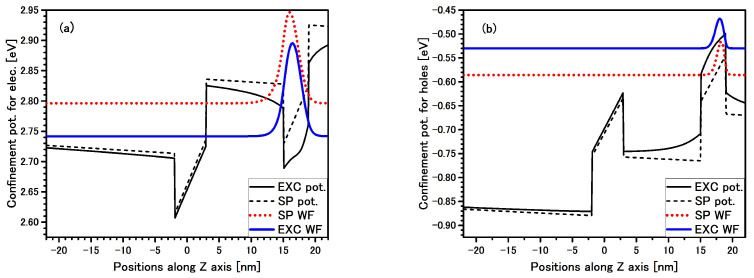
Band edge profile potentials (black lines) with single particle (SP) wave-function (WF) (dotted red line) and excitonic multiplier of the WF (solid blue line) for electron (**a**) and hole (**b**) for direct EL1 exciton in QW 4nm are presented in the structure #1. Solid black lines are confined potential with additional Coulomb term (Equation (7)).

In Figure 2 the electron and hole multipliers of the excitonic WF for direct exciton in QW 4 nm are presented. This is a typical direct exciton, i.e., all WF are well localized in single QW despite an additional Coulomb term lowering the confinement potential for both carriers, and the electric field is applied. In addition, and for comparison, the SP WF of the 8 k·p Hamiltonian with band edge potential is also shown by dashed lines.

**Figure 3:**
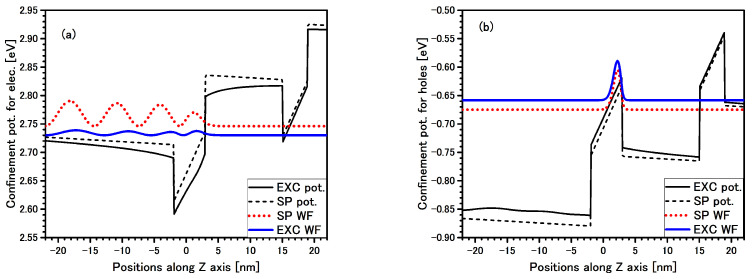
Band edge profile potentials (black lines) with single particle (SP) wave-function (WF) (dotted red line) and excitonic multiplier of the WF (solid blue line) for electron (**a**) and hole (**b**) for extended EL1 exciton in QW 5nm are presented in the structure #1. Solid black lines are confined potential with additional Coulomb term (Equation (7)).

In Figure 3, electron and hole multipliers of the specific type of an exciton which appears in the considered structures are presented. This exciton has built up with SP electron and hole states #1 in QW 5 nm (i.e., this is grand states for the electron and the hole in QW 5 nm) and the electron multiplier of the excitonic WF is extended all over the cap layer with the energy above the barrier. On the other hand, the hole multiplier of the excitonic WF is well localized in the QW 5 nm. This extended electron exciton (ExEX) has about 12 meV binding energy, and the oscillator strength is comparable to the SP transitions. The energy of the ExEX is covered with the FX label (3.4 eV). A very similar situation occurs for ExEX, but with the EL2 multiplier of the excitonic WF.

The PL of localized excitons in the 4 nm well disappears with temperature, and above 120 K it is impossible to observe it on the background of FX emission that is observed as up to 300 K in the case of the 5 nm well.

### 3.2. Structure #2: 4 nm and 5 nm ZnO QWs with 2.5 nm ZnMgO Barriers

The structure that will be studied is formed by two ZnO quantum wells with the same widths (4 and 5 nm) as in the previous structure, the ZnMgO barrier between the QWs is thinner and equal to 2.5 nm. The barrier layer is sufficiently thin to allow charges to the tunnel between the QW layers. Incidentally, the electrons initially created in one of the wells can tunnel to the second well and form a two-dimensional gas of spatially IX. In structure #2, due to a lower concentration of Mg (=9%) in QWs than the previous structure #1 (Mg = 10%), the electric field in QWs has slightly lower value 0.23 MV/cm with the 0.0062 MV/cm strength in the barrier.

**Figure 4:**
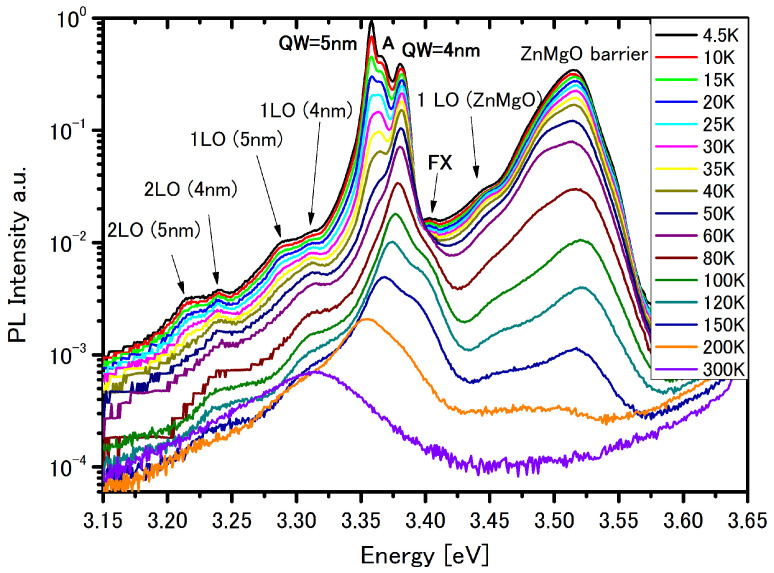
PL spectra at different temperatures for structure #2.

The PL spectra taken at different temperatures for this structure are depicted in Figure 4. The emission peak originating from excitonic transitions in the ZnMgO barrier is observed at 3.52 eV. Emission from the ZnMgO buffer layer is not observed at temperatures above 200 K.

The emission peak observed at 3.358 eV and 3.383 eV is contributed to SP transitions in 5 nm and 4 nm QWs, respectively—Table 3.

The first peak at 3.358 eV is also covered by the direct EL1 exciton (Table 4) but now, the exciton is located in QW 4nm.

**Figure 5:**
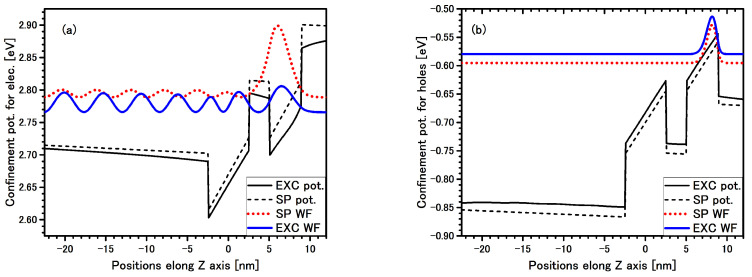
Band edge profile potentials (black lines) with single particle (SP) wave-function (WF) (dotted red line) and excitonic multiplier of the WF (solid blue line) for electron (**a**) and hole (**b**) for direct EL2 exciton in QW 4nm are presented in the structure #2. Solid black lines are confined potential with additional Coulomb term (Equation (7)).

At the 3.367 eV we can see a peak (marked by A) which we identify as the direct EL2 exciton in QW 4 nm—Table 4. The electron and hole multipliers for the direct EL2 exciton are shown in Figure 5, and with a very similar look to the multipliers WF for direct EL1 exciton. It can be seen that the maximum electron multiplier is located in QW 4nm, but due to an additional Coulomb term lowering the confinement potential, there is much stronger leaking out of (tunnelling) the WF than in the SP picture. In addition, because of the thinner barrier width between QWs, lowering of the energy of the electron due to the built-in electric field is smaller than in the case of the previous structure—structure #1. This is the reason why, for structure #2, a transition can be seen at 3.367 eV.

At energy 3.4 eV (marked by FX) for both structures—#1 and #2—the situation is very similar, and this transition energy is attributed to extended EL1 exciton in QW 5 nm (Table 4). This exciton is very similar to that presented in Figure 3—the extended EL1 exciton in structure #1.

### 3.3. Structure #3: 2 nm and 4 nm ZnO QWs with 2.5 nm ZnMgO Barriers

**Figure 6:**
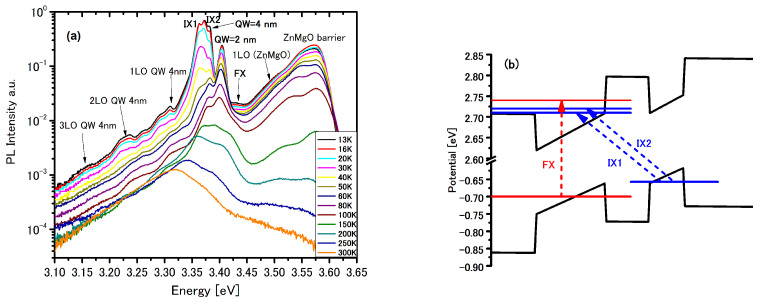
(**a**) The PL spectra at different temperatures for structure #3. (**b**) Schematic diagram for spatially direct (FX—dash red line) and indirect (IX1 and IX2—dash blue lines) excitons in a tilted DQW with narrow ZnMgO barrier. For continence, the confinement potential (black lines) and electron end hole energy levels are in single particle picture.

The first panel in Figure 6 shows PL spectra of the structure with double quantum wells (4 nm and narrow 2 nm) with the barrier 2.5 nm thick—structure #3—within a temperature range of 13–300 K. In this structure, the electric fields in the QWs are very similar to the of structure #2 but with a lower value of 0.0042 MV/cm in the barrier. A broad peak at approx. 3.577 eV is assigned to the ZnMgO layer and 1 LO (3.505 eV). Emission from the ZnMgO buffer layer is observed to room temperature.

The big difference between previous structures (#1 and #2) and this structure #3 is that there are no well-defined electrons in QW 2 nm because of the narrow width of the barrier (2.5 nm) and the narrow width of QW (2 nm). Thus, the strongest SP transitions occur between the ground state electron in QW 4 nm and the first hole (split up into the light (HH1) and heavy (HH2) holes) and the second hole (split into the light (HH3) and heavy (HH4) holes). Even more, the oscillator strength for both of these transitions is very similar to each other—see Table 5.

The experimental peak at 3.382 eV as in all previous cases is again attributed to ground state transitions in QW 4 nm. The peak at 3.405 eV could be aligned to the transition between the first electron and second hole energy levels, also in QW 2 nm.

On the lower energy side of the ground state transitions in QW 4nm we observed two additional peaks at 3.364 eV (marked by IX1) and 3.372 eV (marked by IX2). These two peaks could be attributed to indirect exciton—Table 6. Energies of all these three excitons are presented by schematic diagram on the second panel in Figure 6.

**Figure 7:**
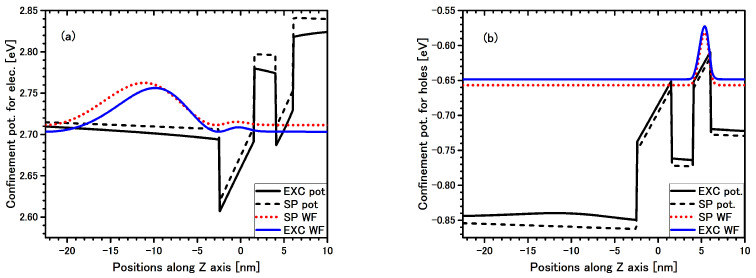
Band edge profile potentials (black lines) with single particle (SP) wave-function (WF) (dotted red line) and excitonic multiplier of the WF (solid blue line) for electron (**a**) and hole (**b**) for indirect EL2 exciton in QW 4 nm are presented in the structure #3. Solid black lines are confined potential with additional Coulomb term (Equation (7)).

The electron and hole multipliers for the indirect EL2 exciton are presented in Figure 7 (for indirect EL3 exciton the electron and hole multipliers are very similar). The electron multiplier for indirect EL2 exciton (Figure 7) in this structure, and for extended EL1 exciton (Figure 3) in structure #1, are both expanded into the cap layer, but the former electron multiplier has only two nodes whereas the latter has many more nodes. It means that the electron factor of the exciton WF is more confined for the indirect than for the direct exciton, so despite the very weak oscillator strength, the transition for the indirect exciton in this structure #3 could be seen.

At 3.431 eV we can see the peak (marked by FX) which, as in structures #1 and #2, we attribute to the extended EL1 exciton in the wider QWs with the energy 3.427 eV—Table 6. In structure #3 the wider QW has a 4 nm width, whereas in structures #1 and #2 it has a 5 nm width, so the FX peak is shifted towards to the higher energy.

## 4. Conclusions

We have carried out a comparative study of asymmetric double QWs structures with different QW widths and with different ZnMgO barriers grown on a–plane ${\mathrm{Al}}_{2}O_{3}$ substrates using PL spectroscopy. The analysis is supported by SP calculation using an 8 k·p wurtzite model in the real geometry of the samples. For the exciton calculation, the 2D hydrogenic 1s–like function for the relative motion of electron and hole particles is applied. The electron and hole multipliers of the exciton WF is calculated using 8 k·p wurtzite, with additional potential which comes from the partial integration of the excitonic WF. All these WF multipliers of the exciton are computed by self-consistent method.

The geometry of structures #1 and #2 are very similar—they only differ in barrier width. The similarity of the PL spectra for these structure—Figure 1 and Figure 4—is confirmed by calculation—by the same SP transitions in both asymmetric QWs (Table 1 and Table 3), and by the same exciton energy (marked in figs by FX label)—extended EL1 exciton (Table 2 and Table 4) which is a direct exciton in the QW 5 nm region. In the structure with the narrow barrier—structure #2—the additional transitions marked by A are observed, and this is the direct EL2 exciton in the QW 4 nm region. Due to interplay between the electric field and barrier width, this type of exciton could not be observed in structure #1—the structure with the thick barrier.

Structure #3 is quite similar to structure #2—it has the same 2.5 nm barrier width and has one QW with the same 4 nm width. The difference between these structures comes from difference in the second QW width—structure #3 has thinner (2 nm width) QW than the adequate QW (5 nm width) in structure #2. Therefore, the SP ground transition in QW 4 nm is in the same energy in all considered structures, and with the left QW in structure #3 having a 4 nm width compared to 5 nm width in other structures, the transition marked by FX comes from the same extended EL1 exciton. Two additional peaks, marked by IX1 and IX2, could be explained by indirect EL2 and indirect EL3 exciton.

## Figures and Tables

**Table 1:** Single particle (SP) energy transitions, oscillator strength both calculated in 8 k·p model and corresponding experimental values for structure #1: 2 QWs with widths 5 and 4 nm and 12 nm barrier between them.

Experimental	Theoretical	Oscillator	Description
Value [eV]	Value [eV]	Strength	
3.358	3.355	0.1828	SP EL1 QW 5 nm to HH1 QW 5 nm
	3.359	0.1711	SP EL1 QW 5 nm to HH2 QW 5 nm
3.383	3.382	0.3492	SP EL1 QW 4 nm to HH1 QW 4 nm
	3.386	0.4270	SP EL1 QW 4 nm to HH2 QW 4 nm

**Table 2:** Calculated excitonic and binding energy, oscillator strength within 8 k·p self-consistent excitonic model for various type of excitons observed in the structure #1.

Excitonic	Binding	Oscillator	Description
Energy [eV]	Energy [meV]	Strength	
3.260	5.854	0.00	Indirect EL1@QW 5 nm–HH1@QW 4 nm
3.328	27.87	0.35	Direct EL1@QW 5 nm–HH1@QW 5 nm
3.347	34.51	0.65	Direct EL1@QW 4 nm–HH1@QW 4 nm
3.409	11.76	0.15	Extended EL1 above QW 5 nm–HH1@QW 5 nm
3.425	13.31	0.17	Extended EL2 above QW 5 nm–HH1@QW 5 nm
3.464	7.58	0.00	Indirect EL1@QW 4 nm–HH1@QW 5 nm

**Table 3:** Single particle (SP) energy transitions, oscillator strength both calculated in 8 k·p model and corresponding experimental values for structure #2: 2 QWs with widths 5 and 4 nm and 2.5 nm barrier between them.

Experimental	Theoretical	Oscillator	Description
Value [eV]	Value [meV]	Strength	
3.358	3.357	0.2116	SP EL1 QW 5 nm to HH1 QW 5 nm
	3.360	0.1571	SP EL1 QW 5 nm to HH2 QW 5 nm
3.383	3.384	0.3750	SP EL1 QW 4 nm to HH1 QW 4 nm
	3.388	0.3171	SP EL1 QW 4 nm to HH2 QW 4 nm

**Table 4:** Calculated excitonic and binding energy, oscillator strength within 8 k·p self-consistent excitonic model for various type of excitons observed in the structure #2.

Excitonic	Binding	Oscillator	Description
Energy [eV]	Energy [meV]	Strength	
3.260	10.40	0.00	Indirect EL1@QW 5 nm–HH1@QW 4 nm
3.329	27.46	0.35	Direct EL1@QW 5 nm–HH1@QW 5 nm
3.352	14.90	0.32	Direct EL1@QW 4 nm–HH1@QW 4 nm
3.365	19.04	0.15	Direct EL2@QW 4 nm–HH1@QW 4 nm
3.404	12.01	0.16	Extended EL1 above QW 5 nm–HH1@QW 5 nm
3.420	13.52	0.17	Extended EL2 above QW 5 nm–HH1@QW 5 nm
3.454	16.35	0.01	Indirect EL1@QW 4 nm–HH1@QW 5 nm

**Table 5:** Single particle (SP) energy transitions, oscillator strength both calculated in 8 k·p model and corresponding experimental values for structure #3: 2 QWs with widths 2 and 4 nm and 2.5 nm barrier between them.

Experimental	Theoretical	Oscillator	Description
Value [eV]	Value [meV]	Strength	
3.382	3.380	0.3839	SP EL1 QW 4 nm to HH1 QW 4 nm
	3.384	0.3754	SP EL1 QW 4 nm to HH2 QW 4 nm
3.405	3.413	0.3508	SP EL1 QW 2 nm to HH1 QW 2 nm
	3.417	0.3413	SP EL1 QW 2 nm to HH2 QW 2 nm

**Table 6:** Calculated excitonic and binding energy, oscillator strength within 8 k·p self-consistent excitonic model for various type of excitons observed in the structure #3.

Excitonic	Binding	Oscillator	Description
Energy [eV]	Energy [meV]	Strength	
3.324	13.45	0.00	Indirect EL1@QW 4 nm–HH1@QW 2 nm
3.347	33.10	0.488	Direct EL1@QW 4 nm–HH1@QW 4 nm
3.361	6.856	0.001	Indirect EL2@QW 4 nm–HH1@QW 2 nm
3.372	6.796	0.001	Indirect EL3@QW 4 nm–HH1@QW 2 nm
3.399	15.34	0.397	Direct EL1@QW 2 nm–HH1@QW 2 nm
3.413	19.36	0.260	Direct EL2@QW 2 nm–HH1@QW 2 nm
3.427	11.18	0.160	Extended EL1 above QW 4 nm–HH1@QW 4 nm
3.443	14.10	0.111	Extended EL2 above QW 4 nm–HH1@QW 4 nm
3.474	14.88	0.081	Indirect EL1@QW 2 nm–HH1@QW 4 nm

## Abbreviations

The following abbreviations are used in this manuscript:

QW	Quantum well
WF	Wave-function
SP	Single particle
IX	Indirect exciton
PL	Photoluminescence
ExEX	Extended electron exciton
